# Supplementary material for: A Retrospective, Single-Center Analysis of Specialized Palliative Care Services for Patients with Advanced Small-Cell Lung Cancer
Source: Cancers (Basel). 2022 Oct 12;14(20):4988. doi: 10.3390/cancers14204988 (PMC9599531; doi:10.3390/cancers14204988)
Supplement: Supplementary file 1 [file cancers-14-04988-s001.zip › cancers-1915440-supplementary.pdf]

Supplemental material

Supplemental Table S1. Comparision of patients' characteristics among the four cutoff values.

	Cutoff a<br>n = 42	Cutoff b<br>n = 33	Cutoff c<br>n = 40	Cutoff d<br>n = 25	*p-value*
Age (years), median (range)	69 (39 – 88)	65 (39 – 85)	68 (39 – 88)	65 (39 – 85)	n.s.*
Male, n (%)	21 (50)	17 (51.5)	22 (55)	11 (44)	n.s.†
Female, n (%)	21 (50)	16 (48.5)	18 (45)	14 (56)	n.s.†
UICC stage at diagnosis:					
- Stage III, n (%)	5 (11.9)	6 (18.2)	6 (15)	4 (16)	n.s.‡
- Stage IV, n (%)	37 (88.1)	27 (81.8)	34 (85)	21 (84)	n.s.†
History of smoking, n (%)	42 (100)	33 (100)	40 (100)	25 (100)	n.s.†
PD-L1 ≥ 1%, n (%)	9 (21.4)	9 (27.3)	11 (27.5)	9 (36)	n.s.‡
ECOG ≥ 2 at time of diagnosis, n (%) (n = 116)	12 (28.6) [a,b,c]	5 (15.2) [a]	6 (15) [b]	5 (20) [c]	0.002‡
Metastatic disease at time of diagnosis:					
- Bone, n (%)	12 (28.6)	11 (33.3)	13 (32.5)	8 (32)	n.s.‡
- Liver, n (%)	18 (42.9)	11 (33.3)	14 (35)	7 (28)	n.s.‡
- Lungs, n (%)	4 (9.5)	4 (12.1)	5 (12.5)	2 (8)	n.s.‡
- Pleura, n (%)	8 (19)	4 (12.1)	7 (17.5)	4 (16)	n.s.‡
- Brain, n (%)	12 (28.6) [a,b,c]	15 (45.5) [a]	17 (42.5) [b]	11 (44) [c]	0.019†
- Extrathoracic lymph nodes, n (%)	8 (19)	7 (21.2)	6 (15)	6 (24)	n.s.‡
- Adrenal glands, n (%)	13 (33.3)	12 (36.4)	15 (37.5)	9 (36)	n.s.†
Therapeutic approaches (first line):					
- Surgery, n (%)	2 (4.8)	2 (6.1)	2 (5)	1 (4)	n.s.‡
- Radio-chemotherapy, n (%)	0	0	0	0	-
- Radiotherapy, n (%)	1 (2.4)	0	0	0	-
- Systemic antineoplastic therapy, n(%)	31 (73.8) [a,b,c]	29 (87.9) [a]	36 (90) [b]	22 (88) [c]	<0.001†
- Best supportive care, n (%)	8 (19) [a,b,c]	2 (6.1) [a]	2 (5) [b]	2 (8) [c]	<0.001‡
Referral indications:					
- Pain, n (%)	10 (23.8)	10 (30.3)	9 (22.5)	7 (28)	n.s.‡
- Dyspnea, n (%)	7 (16.7)	3 (9.1)	5 (12.5)	3 (12)	n.s.‡
- Other symptoms, n (%)	3 (7.1)	3 (9.1)	7 (17.5)	3 (12)	‡
- Pain and additional other symptoms, n (%)	1 (2.4)	0	1 (2.5)	0	-
- Dying patient, n (%)	2 (4.8)	0	0	0	-
- Social aspects, n (%)	1 (2.4)	3 (9.1)	3 (7.5)	2 (8)	n.s.‡
- General integration of PC, n (%)	18 (42.9)	14 (42.4)	15 (37.5)	10 (40)	n.s.†
Duration of SPC services (days), median (range)	168 (1 – 1876)	74 (1 – 1876)	164 (27 – 956)	248 (129 – 956)	n.s.*
Time from diagnosis till start of SPC (days), median (range)	6 (0 – 61) [a,b,c]	32 (0 – 1315) [a]	10 (0 – 1315) [b]	8 (0 – 643) [c]	< 0.001#

* One-way ANOVA was applied, #Kruskal-Wallis test was applied, †Chi-square test was applied, ‡Fisher Exact test was applied. [a] $p < 0.05$ cutoff a vs. cutoff b,[b] $p < 0.05$ cutoff a vs. cutoff c, [c] $p < 0.05$ cutoff a vs. cutoff d, UICC = Union Internationale Contre le Cancer, PD-L1 = programmed death-ligand 1, ECOG = Eastern Cooperative Oncology Group, SPC = specialized palliative care, PC = palliative care.

Supplemental Table S2. Comparision of patients' characteristics of early and late referral for SPC according to cutoff a.

Cutoff a (SPC < 60 days after diagnosis)			
	**Early (n = 42)**	**Late (n = 33)**	***p-value***
Age (years), median (range)	69 (39 – 88)	65 (46 – 86)	n.s.†
Male, n (%)	21 (50)	24 (72.7)	n.s.*
Female, n (%)	21 (50)	9 (27.3)	n.s.#
UICC stage at diagnosis:			
- Stage III, n (%)	5 (11.9)	7 (21.2)	n.s.#
- Stage IV, n (%)	37 (88.1)	26 (78.8)	n.s.*
History of smoking, n (%)	42 (100)	30 (90.9)	n.s.*
PD-L1 ≥ 1%, n (%)	9 (21.4)	6 (18.2)	n.s.#
ECOG ≥ 2 at time of diagnosis, n (%) (n = 63)	12 (35.3)	2 (6.9)	0.007#
Metastatic disease at time of diagnosis:			
- Bone, n (%)	12 (28.6)	8 (24.2)	n.s.#
- Liver, n (%)	18 (42.9)	12 (36.4)	n.s.*
- Lungs, n (%)	4 (9.5)	4 (12.1)	n.s.#
- Pleura, n (%)	8 (19)	1 (3)	n.s.#
- Brain, n (%)	12 (28.6)	16 (48.5)	n.s.*
- Extrathoracic lymph nodes, n (%)	8 (19)	5 (15.2)	n.s.#
- Adrenal glands, n (%)	14 (33.3)	9 (27.3)	n.s.#
Therapeutic approaches (first line):			
- Surgery, n (%)	2 (4.6)	0	n.s.#
- Radiotherapy, n (%)	1 (2.4)	1 (3)	n.s.#
- Systemic antineoplastic therapy, n(%)	31 (73.9)	31 (93.9)	n.s.*
- Best supportive care, n (%)	8 (19)	1 (3)	n.s.#
Referral indications:			
- Pain, n (%)	10 (23.8)	11 (33.3)	n.s.#
- Dyspnea, n (%)	7 (16.7)	1 (3)	n.s.#
- Other symptoms, n (%)	3 (7.1)	12 (36.4)	0.003#
- Pain and additional other symptoms, n (%)	1 (2.4)	3 (9.1)	n.s.#
- Dying patient, n (%)	2 (4.8)	1 (3)	n.s.#
- Social aspects, n (%)	1 (2.4)	3 (9.1)	n.s.#
- General integration of PC, n (%)	18 (42.9)	2 (6.1)	< 0.001#
Duration of SPC services (days), median (range)	168 (1 – 1876)	31 (1 – 752)	0.016†
Time from diagnosis till start of SPC (days), median (range)	5.5 (1 – 61)	302 (46 – 1315)	< 0.001†
Survival time (months), median (range)	5 (0 – 61)	12 (0 – 44)	0.007‡

†Mann–Whitney-U test was applied, *chi-square test was applied, #Fisher's exact test was applied, ‡log-rank test was applied. UICC = Union Internationale Contre le Cancer, PD-L1 = Programmed death-ligand 1, ECOG = Eastern Cooperative Oncology Group, SPC = Specialized palliative care.

Supplemental Table S3. Comparision of patients' characteristics of early and late referral for SPC according to cutoff b.

Cutoff b (≥ 60 days before death)			
	**Early (n = 33)**	**Late (n = 32)**	***p-value***
Age (years), median (range)	65 (39 – 85)	67 (46 – 88)	n.s.†
Male, n (%)	17 (51.5)	24 (75)	n.s.*
Female, n (%)	16 (48.5)	8 (25)	n.s.#
UICC stage at diagnosis:			
- Stage III, n (%)	6 (18.2)	4 (12.5)	n.s.#
- Stage IV, n (%)	27 (81.8)	28 (87.5)	n.s.*
History of smoking, n (%)	33 (100)	30 (93.8)	n.s.*
PD-L1 ≥ 1%, n (%)	9 (27.3)	5 (15.6)	n.s.#
ECOG ≥ 2 at time of diagnosis, n (%) (n = 54)	5 (17.9)	4 (15.4)	n.s.#
Metastatic disease at time of diagnosis:			
- Bone, n (%)	11 (33.3)	9 (28.1)	n.s.#
- Liver, n (%)	11 (33.3)	16 (50)	n.s.*
- Lungs, n (%)	4 (12.1)	1 (3.1)	n.s.#
- Pleura, n (%)	8 (19)	1 (3)	n.s.#
- Brain, n (%)	15 (45.5)	11 (34.4)	n.s.*
- Extrathoracic lymph nodes, n (%)	7 (21.2)	3 (9.4)	n.s.#
- Adrenal glands, n (%)	10 (23.8)	11 (33.3)	n.s.#
Therapeutic approaches (first line):			
- Surgery, n (%)	2 (6.1)	0	n.s.#
- Radiotherapy, n (%)	0	1 (3.1)	n.s.#
- Systemic antineoplastic therapy, n(%)	29 (87.9)	25 (78.1)	n.s.*
- Best supportive care, n (%)	2 (6.1)	6 (18.8)	n.s.#
Referral indications:			
- Pain, n (%)	10 (30.3)	9 (28.1)	n.s.#
- Dyspnea, n (%)	3 (9.1)	5 (15.6)	n.s.#
- Other symptoms, n (%)	3 (9.1)	10 (31.3)	0.033#
- Pain and additional other symptoms, n (%)	1 (2.4)	3 (9.1)	n.s.#
- Dying patient, n (%)	0	3 (9.4)	n.s.#
- Social aspects, n (%)	3 (9.1)	1 (3.1)	n.s.#
- General integration of PC, n (%)	14 (42.4)	2 (6.3)	< 0.001#
Duration of SPC services (days), median (range)	200 (60 – 965)	15 (1 – 59)	< 0.001†
Time from diagnosis till start of SPC (days), median (range)	8 (1 – 643)	117 (1 – 1315)	0.012†
Survival time (months), median (range)	8 (2 – 46)	3 (0 – 44)	0.025‡

†Mann–Whitney-U test was applied, *chi-square test was applied, #Fisher's exact test was applied, ‡log-rank test was applied. UICC = Union Internationale Contre le Cancer, PD-L1 = Programmed death-ligand 1, ECOG = Eastern Cooperative Oncology Group, SPC = Specialized palliative care.

Supplemental Table S4. Comparision of patients' characteristics of early and late referral for SPC according to cutoff c.

Cutoff c (≥ 30 days before death)			
	**Early (n = 40)**	**Late (n = 25)**	***p-value***
Age (years), median (range)	68.5 (39 – 88)	63.5 (46 – 77)	n.s.†
Male, n (%)	22 (55)	19 (76)	n.s.*
Female, n (%)	18 (45)	6 (24)	n.s.#
UICC stage at diagnosis:			
- Stage III, n (%)	6 (15)	4 (16)	n.s.#
- Stage IV, n (%)	34 (85)	21 (84)	n.s.*
History of smoking, n (%)	40 (100)	23 (92)	n.s.*
PD-L1 ≥ 1%, n (%)	11 (27.5)	3 (12)	n.s.#
ECOG ≥ 2 at time of diagnosis, n (%) (n = 54)	6 (17.6)	3 (15)	n.s.#
Metastatic disease at time of diagnosis:			
- Bone, n (%)	13 (32.5)	7 (28)	n.s.#
- Liver, n (%)	14 (35)	13 (52)	n.s.*
- Lungs, n (%)	5 (12.5)	0	n.s.#
- Pleura, n (%)	7 (17.5)	2 (8)	n.s.#
- Brain, n (%)	17 (42.5)	9 (36)	n.s.#
- Extrathoracic lymph nodes, n (%)	6 (15)	4 (16)	n.s.#
- Adrenal glands, n (%)	15 (37.5)	6 (24)	n.s.#
Therapeutic approaches (first line):			
- Surgery, n (%)	2 (5)	0	n.s.#
- Radiotherapy, n (%)	0	1 (4)	n.s.#
- Systemic antineoplastic therapy, n(%)	36 (90)	18 (72)	n.s.*
- Best supportive care, n (%)	2 (5)	6 (24)	n.s.#
Referral indications:			
- Pain, n (%)	9 (22.5)	10 (40)	n.s.#
- Dyspnea, n (%)	5 (12.5)	3 (12)	n.s.#
- Other symptoms, n (%)	7 (17.5)	6 (24)	n.s.#
- Pain and additional other symptoms, n (%)	1 (2.5)	1 (4)	n.s.#
- Dying patient, n (%)	0	3 (12)	n.s.#
- Social aspects, n (%)	3 (7.5)	1 (4)	n.s.#
- General integration of PC, n (%)	15 (37.5)	1 (4)	0.002#
Duration of SPC services (days), median (range)	164.5 (31 – 965)	11 (1 – 29)	< 0.001†
Time from diagnosis till start of SPC (days), median (range)	10 (1 – 1315)	191(1 – 647)	0.038†
Survival time (months), median (range)	8 (0 – 46)	6 (0 – 21)	0.025‡

†Mann–Whitney-U test was applied, *chi-square test was applied, #Fisher's exact test was applied, ‡log-rank test was applied. UICC = Union Internationale Contre le Cancer, PD-L1 = Programmed death-ligand 1, ECOG = Eastern Cooperative Oncology Group, SPC = Specialized palliative care.

Supplemental Table S5. Comparision of patients' characteristics of early and late referral for SPC according to cutoff d.

Cutoff d (≥ 120 days before death)			
	**Early (n = 25)**	**Late (n = 40)**	***p-value***
Age (years), median (range)	65 (39 – 85)	67 (46 – 88)	n.s.†
Male, n (%)	11 (44)	30 (75)	n.s.*
Female, n (%)	14 (56)	10 (25)	n.s.#
UICC stage at diagnosis:			
- Stage III, n (%)	4 (16)	6 (15)	n.s.#
- Stage IV, n (%)	21 (84)	34 (85)	n.s.*
History of smoking, n (%)	25 (100)	38 (95)	n.s.*
PD-L1 ≥ 1%, n (%)	9 (36)	5 (12.5)	n.s.#
ECOG ≥ 2 at time of diagnosis, n (%) (n = 54)	5 (22.7)	4 (12.5)	n.s.#
Metastatic disease at time of diagnosis:			
- Bone, n (%)	8 (32)	12 (30)	n.s.#
- Liver, n (%)	7 (28)	20 (50)	n.s.#
- Lungs, n (%)	2 (8)	3 (7.5)	n.s.#
- Pleura, n (%)	4 (16)	5 (12.5)	n.s.#
- Brain, n (%)	11 (44)	15 (37.5)	n.s.#
- Extrathoracic lymph nodes, n (%)	6 (24)	4 (10)	n.s.#
- Adrenal glands, n (%)	9 (36)	12 (30)	n.s.#
Therapeutic approaches (first line):			
- Surgery, n (%)	1 (4)	1 (2.5)	n.s.#
- Radiotherapy, n (%)	0	1 (2.5)	n.s.#
- Systemic antineoplastic therapy, n(%)	22 (88)	32 (80)	n.s.*
- Best supportive care, n (%)	2 (8)	6 (15)	n.s.#
Referral indications:			
- Pain, n (%)	7 (28)	12 (30)	n.s.#
- Dyspnea, n (%)	3 (12)	5 (12.5)	n.s.#
- Other symptoms, n (%)	3 (12)	10 (25)	n.s.#
- Pain and additional other symptoms, n (%)	0	2 (5)	n.s.#
- Dying patient, n (%)	0	3 (7.5)	n.s.#
- Social aspects, n (%)	2 (8)	2 (5)	n.s.#
- General integration of PC, n (%)	10 (40)	5 (15)	0.037#
Duration of SPC services (days), median (range)	248 (129 – 965)	18 (1 – 98)	< 0.001†
Time from diagnosis till start of SPC (days), median (range)	8 (1 – 643)	73 (1 – 1315)	n.s.†
Survival time (months), median (range)	9 (4 – 46)	3 (0 – 44)	0.003‡

†Mann–Whitney-U test was applied, *chi-square test was applied, #Fisher's exact test was applied, ‡log-rank test was applied. UICC = Union Internationale Contre le Cancer, PD-L1 = Programmed death-ligand 1, ECOG = Eastern Cooperative Oncology Group, SPC = Specialized palliative care.
